# Volitional Inhibition and Brain–Machine Interfaces: A Mandatory Wedding

**DOI:** 10.3389/fneng.2012.00020

**Published:** 2012-08-28

**Authors:** Giovanni Mirabella

**Affiliations:** ^1^Istituiti di Ricovero e Cura a Carattere Scientifico NeuromedPozzilli, Italy; ^2^Department of Physiology and Pharmacology “V. Erspamer,” La Sapienza UniversityRome, Italy

A key feature of voluntary behavior is its flexibility which, in a sense, represents the other side of self-control. We need to select and perform actions whenever they are more opportune, i.e., whenever the costs intrinsically associated with them are lower than their benefits. Given that we cannot predict with certainty the occurrence of an event and the time lag elapsing between the decision to move and the physical execution of a movement, we have developed the ability to cancel pending actions. Suppressing ongoing acts is fundamental when sudden changes in the surrounding environment take place. For instance, the sudden arrival of a car in the road we were about to cross requires us to stop our step to avoid being hit. The great importance of this executive function, named “volitional” inhibition, is witnessed by the great number of brain regions implicated in its elaboration. Here, the term volitional does not imply a conscious participation. In humans the emergence of awareness coupled with the vetoing ability gave rise to what Libet (1985) called “free would not,” that is, our capacity to freely cancel those actions we do not wish to perform. However, we do not exert our free will on every choice we have to make, but just on those more controversial or salient (e.g., how to respond to the request of working over the week-end). Most of the time the fate of actions is decided by automatic processes, otherwise we could not have sufficient free capacity for other computations.

Overall volitional inhibition represents a cornerstone of voluntary behavior but, despite an incredible amount of work, both the localizations of its neural substrates and their specific contributions are still controversial. For instance, it has been suggested that inhibitory commands are generated in a right-lateralized frontal–basal ganglia–thalamic network (Aron et al., 2007), but there is scant knowledge about where they act. One paper in this Research Topic (Mattia et al., 2012) indicates that the motor cortices (both the primary motor cortex and the premotor cortex) are the targets of cancelation commands (see also Mirabella et al., 2011). In other words, it suggests that the same neural substrates involved in planning and executing an act (see also Busan et al., 2012) are also involved in its suppression. Along the same lines, Pastor-Bernier et al. (2012) show that neurons of the premotor cortex continuously update their activities during movement planning, so that their discharge reflects switches between alternative plans when a selected movement option suddenly turns out to be inappropriate.

Importantly, as described in the review by Stuphorn and Emeric (2012), the inhibitory network not only provides signals indicating the withholding of actions whenever a stop signal is presented (reactive control), but also provides signals enabling the subject to adopt a response strategy which takes into account the context in which he or she operates. This form of cognitive control over response execution, named proactive control, adjusts the response selection and preparation process in anticipation of known task demands, driven by endogenous signals. Several lines of evidence indicate the involvement of the medial frontal cortex in proactive control. Wriessnegger et al. (2012) also ascribe to these regions a role in inhibiting learned motor programs.

Despite the relevance of volitional inhibition in shaping voluntary behavior, its role has been almost completely neglected by those scientists who tried to implement brain–machine interfaces (BMIs). These interfaces are aimed at exploiting neural activity recorded from the brain, e.g., motor commands, to control the movements of external devices, e.g., prosthetic limbs. By translating brain signals into action, a BMI can enable a person suffering from paralysis to move again using artificial limbs. Notwithstanding the successes of the BMI approach there are still several limitations. Signals extracted from the brain are typically noisy, so the guidance of prosthetic limbs is still far from approximating natural behaviors. In this Research Topic, three papers report significant improvements in BMI algorithms. Wang et al. (2012) demonstrate the possibility of detecting the onset and the direction of intended movements exploiting electrocorticographic signals recorded from the surface of the cortex of pharmacoresistant epileptic patients. Lew et al. (2012) show that from the analysis of the readiness potential is possible to detect the movement intention in single trials. This is very relevant because the capability of detecting the neural correlates of self-paced movement at the single-trial level is a fundamental step toward the development of efficient BMIs. In fact, normally we perform a given movement just once rather than repeating them several times in a stereotyped fashion. As a consequence, BMI algorithms relying on brain activity averaged over a number of trials are not very naturalistic.

Finally, Ifft et al. (2012) successfully tried, for the first time, to implement a BMI that can extract response inhibition signals and thus can mimic the suppression of a motor plan and its reprogramming when required by external events. As a model they used single-unit activity of over trained monkeys so it is not obvious that this result could be applied effortlessly to humans; however, it is the first demonstration that brain signals sustaining the flexibility of human behavior can be fed into a BMI. In the same direction, Yang et al. (2012) have developed a field-programmable gate array using dedicated real-time hardware circuitry exploiting a model built on the firing rate recorded in the frontal eye field of monkeys during a countermanding oculomotor task (a task which probes the subject’s ability to stop pending saccades). Their device is able to simulate the behavioral performance during the task, showing the reliability of the inhibitory control system that can potentially be employed to build an efficient prosthetic system. Although intriguing, further studies are required to assess whether this system can be generalized to limb movements, as there is evidence showing that the saccadic and arm movements are controlled in different ways (see, e.g., Mirabella et al., 2009, 2011).

All in all I hope that the readers of this issue will be convinced of intimate and inextricable connection between volitional inhibition and voluntary behavior. From this it should naturally follow that, in order to allow prosthetic devices to mimic naturally enacted movements, it is necessary to build algorithms capable of decoding brain activity underlying the suppression of pre-programmed actions when unpredictable events require a quick change of the planned motor strategy.
